# Author Correction: *Gene.iobio*: an interactive web tool for versatile, clinically-driven variant interrogation and prioritization

**DOI:** 10.1038/s41598-022-09959-3

**Published:** 2022-04-06

**Authors:** Tonya Di Sera, Matt Velinder, Alistair Ward, Yi Qiao, Stephanie Georges, Chase Miller, Anders Pitman, Will Richards, Aditya Ekawade, David Viskochil, John C. Carey, Laura Pace, Jim Bale, Stacey L. Clardy, Ashley Andrews, Lorenzo Botto, Gabor Marth

**Affiliations:** University of Utah, Salt Lake City, UT USA

Correction to: *Scientific reports* 10.1038/s41598-021-99752-5, published online 13 October 2021

The Competing interests section in the original version of this Article was incorrect.

“The authors declare no competing interests.”

now reads:

“Gabor Marth is a founder and chief scientific officer of Frameshift Labs, Inc. and Alistair Ward is a founder and chief executive officer of Frameshift Labs, Inc.”

The original Article has been corrected.

